# Comparison of pharmacist managed anticoagulation with usual medical care in a family medicine clinic

**DOI:** 10.1186/1471-2296-12-88

**Published:** 2011-08-17

**Authors:** Stephanie Young, Lisa Bishop, Laurie Twells, Carla Dillon, John Hawboldt, Patrick O'Shea

**Affiliations:** 1 School of Pharmacy, Memorial University, 300 Prince Philip Drive, St. John's, A1B 3V6, Canada; 2 Faculty of Medicine, Memorial University, 300 Prince Philip Drive, St. John's, A1B 3V6, Canada; 3 Newfoundland Drive Family Practice, 427 Newfoundland Drive, St. John's, A1A 4A5, Canada

## Abstract

**Background:**

The beneficial outcomes of oral anticoagulation therapy are dependent upon achieving and maintaining an optimal INR therapeutic range. There is growing evidence that better outcomes are achieved when anticoagulation is managed by a pharmacist with expertise in anticoagulation management rather than usual care by family physicians. This study compared a pharmacist managed anticoagulation program (PC) to usual physician care (UC) in a family medicine clinic.

**Methods:**

A retrospective cohort study was carried out in a family medicine clinic which included a clinical pharmacist. In 2006, the pharmacist assumed anticoagulation management. For a 17-month period, the PC group (n = 112) of patients on warfarin were compared to the UC patients (n = 81) for a similar period prior to 2006. The primary outcome was the percentage of time patients' INR was in the therapeutic range (TTR). Secondary outcomes were the percentage of time in therapeutic range within ± 0.3 units of the recommended range (expanded TTR) and percentage of time the INR was >5.0 or <1.5.

**Results:**

The baseline characteristics were similar between the groups. Fifty-five percent of the PC group was male with a mean age of 67 years; 51% of the UC group was male with a mean age of 71 years. The most common indications for warfarin in both groups were atrial fibrillation, mechanical heart valves and deep vein thrombosis. The TTR was 73% for PC and 65% for UC (p < 0.0001). The expanded TTR for PC was 91% and 85% for UC (p < 0.0001). The percentage of time INR values were <1.5 was 0.7% for PC patients and 1.9% for UC patients (p < 0.0001), and >5 were 0.3% for PC patients and 0.1% for UC (p < 0.0001).

**Conclusion:**

The pharmacist-managed anticoagulation program within a family practice clinic compared to usual care by the physicians achieved significantly better INR control as measured by the percentage of time patients' INR values were kept in both the therapeutic and expanded range. Based on the results of this study, a collaborative family practice clinic using pharmacists and physicians may be an effective model for anticoagulation management with these results verified in future prospective randomized studies.

## Background

Warfarin has been the main oral anticoagulant used for more than six decades, and its effectiveness for the prevention and treatment of thromboembolism is well established [1]. The effectiveness and safety of warfarin is dependent on maintaining the international normalized ratio (INR) in a narrow therapeutic range. This is challenging in clinical practice due to factors including diet and concurrent medications which may alter the pharmacokinetics of warfarin, and therefore the stability of the INR [2].

Newer oral anticoagulants are available in Canada with more predictable pharmacologic properties than warfarin thus eliminating the need for frequent monitoring [3,4]. Ongoing and completed clinical trials with the new agents will likely expand their usage [5-8]. However, there may continue to be a need for warfarin, in patient populations not studied in clinical trials or who are unable to receive the newer medications due to adverse effects or contraindications. Consequently, there will be an ongoing need for practitioners with an expertise in anticoagulation management to provide the necessary level of care to patients still requiring warfarin.

In Canada, patients taking warfarin are usually managed by their family physician in a clinic setting. The current recommendation for health care providers managing oral anticoagulation, is to do so in a "systematic and coordinated fashion, incorporating patient education, systematic INR testing, tracking, follow-up and good patient communication" [1]. The complexity of managing oral anticoagulation has led to the development of a variety of specialized care models including patient self-management, specialized anticoagulation clinics and pharmacist managed services.

Both observational and randomized controlled trials have compared anticoagulation management provided by a pharmacist led anticoagulation clinic versus usual physician care [9-15]. Some of these studies showed improvement in anticoagulation management by pharmacists while others have found no difference as measured by the percentage of time INR was in the therapeutic range (TTR). Pharmacist-staffed anticoagulation clinic models point to more consistent monitoring, use of warfarin dosage adjustment algorithms, early recognition of patient risk factors, and patient education as the mechanisms by which they achieve better outcomes than were being achieved through traditional models of care [9-13]. There is some evidence to suggest that a pharmacist within a family practice clinic encourages collaboration with the physicians, which improves continuity and integration of care [16]. The current literature does not indicate any Canadian centres that have examined anticoagulation management by a pharmacist who was a team member in a family medicine clinic.

A clinical pharmacist with expertise in anticoagulation management was incorporated into a family medicine clinic in St. John's, NL. As part of this role, the pharmacist managed the anticoagulation of all clinic patients taking warfarin using an evidenced-based protocol [1,2,17,18]. We conducted a retrospective cohort study to compare anticoagulation control by the pharmacist to that provided by the family physicians.

## Methods

### Study design and setting

We conducted a retrospective cohort study to compare the anticoagulation control of pharmacist managed (PC) oral anticoagulation to usual care (UC) provided by family physicians. The family medicine clinic is located in St. John's, NL, a capital city with a population of approximately 187 000 [19]. At the time of the study the clinic was comprised of four to five full-time family physicians and two part time family physicians, and is affiliated with Memorial University. The pharmacist joined the clinic in December 2005 and as part of the position in December 2006 assumed day to day anticoagulation management of clinic patients receiving warfarin.

### Models of Care

#### Usual Care

The usual care model by the family physicians (prior to pharmacist management) generally required patients to have blood drawn by venipuncture for an INR test, and processed at the local hospital laboratory. The patient was requested to notify the physician's office that the test had been completed. The results were retrieved from the computerized laboratory system by the physician (or clinic staff and distributed to the physician), most often the same day as the test was completed. Each individual physician assessed the result for their patients, and either called their patient directly via telephone, or had the clinic staff call via telephone, to inform the patient of the results and changes if required. Late day results were handled by the clinic on-call physician. Dosage changes and time intervals for INR blood tests, as well as warfarin related education were at the discretion of the individual physician. Depending on the indication for warfarin, and whether the patient had been admitted to hospital, they may have received anticoagulation related education prior to presenting to the family physician for management. No specific dosing nomogram was utilized; individual physician knowledge and experience with management of warfarin was utilized. Changes to warfarin dosing and schedule for next INR were recorded in the patient's chart. Most often, the patient's usual family physician assessed the results; however, when their own physician was unavailable, another clinic physician completed the assessment and management.

#### Pharmacist Care

The pharmacists involved in the anticoagulation management had completed a Bachelor's degree in Pharmacy, a Doctor of Pharmacy (Pharm.D.), and two specific, short (<1 to 6 week) courses in anticoagulation management. Prior to the pharmacist assuming management of the warfarin therapy, a clinic-specific protocol was developed by the pharmacist based on current guidelines and were approved by the clinic physicians [1,2,17,18]. The format and content of the protocol followed the recommendations for delivering optimized anticoagulation therapy in an outpatient setting [17]. This protocol included evidence-based guidelines regarding dosage recommendations and intervals for INR testing, but allowed flexibility for the pharmacist to assess each patient individually to develop patient-specific recommendations. Similarly to the physician model, the pharmacist care model required the patient to have blood drawn for an INR test, usually by venipuncture and processed at the local hospital laboratory, and patients were requested notify either the physician's office or the pharmacist that the test had been completed. Patients were managed primarily via telephone, with home or clinic visits as needed for initial education. The pharmacist maintained a list of all the clinic patients who were receiving warfarin therapy, and a daily log of patients scheduled for INR testing. The pharmacist was responsible for retrieving the INR test results daily (weekdays) from the computerized laboratory system, and following up with patients as needed who did not complete scheduled testing. Late day results were handled by the pharmacist. Once a test result was retrieved, the pharmacist telephoned the patient (or caregiver) and assessed patients for factors that would affect the result and/or the subsequent recommendation: i.e., changes in medications or diet, signs and symptoms of haemorrhagic or thromboembolic events, missed doses and illnesses. The pharmacist used the protocol guideline as well as clinical judgement/specialized knowledge to develop the care plan of dosage change if required and follow up INR testing, and provided patient counselling. Documentation of assessment and recommendations was made directly into the patient's chart, and available to the physicians. The physicians were readily accessible to the pharmacist for discussion of patient related issues. The clinic protocol outlined mandatory physician contact by the pharmacist for discussion of management (i.e., INR>5.0, suspicion of serious adverse effects, new clots or serious bleeding). Patients had the ability to contact either the physician or the pharmacist for anticoagulation related questions or concerns.

### Data abstraction

A pharmacy student used a data abstraction form to abstract data from the regional health authority's computerized database and family physician and pharmacist's written and electronic records. Patient demographics, indications for warfarin, INR target and duration of therapy and risk factors for bleeding or thromboembolic events were obtained from the physician (UC group) or pharmacist (PC group) records. Results of INR testing, emergency room visits or inpatient admissions, and verification of major bleeding and thromboembolic events were obtained from the regional health authority database. A sample of completed data collection forms were verified by two investigators to ensure the validity and reliability of data. Data were entered into a web based program (ClotFree System, Genesis Advanced Technologies, Inc, Lakehills, TX), designed for routine warfarin monitoring and patient maintenance. Permission was granted to utilize the database for this study using coded patient information.

### Eligibility criteria

Patients eligible for inclusion were those ≥ 19 years, required warfarin for at least 3 months, had at least two INR values not more than 6 weeks apart and were patients of the clinic. The INR values used were those noted in the patients' records. All eligible patients whose warfarin was managed by the pharmacist from December 7, 2006 to May 7, 2008 were included in the PC group. The UC group comprised eligible patients whose warfarin was managed by a clinic physician between July 6, 2005 up to December 6, 2006. INR values were excluded from the data if they were within the first 30 days of warfarin initiation or hospital discharge, during hospitalization, or during temporary planned interruptions, which was considered between the first day warfarin was held and two weeks after warfarin was restarted [18]. These are timeframes of potential INR instability, and during this time they may also be outside the control of either the pharmacist or the family physician.

### Study outcomes

The primary study outcome was to compare anticoagulation control by the pharmacist to that provided by the family physicians as measured by the percentage of time patients' INR was within the recommended therapeutic range (TTR). The recommended therapeutic range was either 2.0 to 3.0 or 2.5 to 3.5, as determined by the indication for anticoagulant therapy [1]. Secondary outcomes were the percentage of TTR within ± 0.3 units of the recommended therapeutic range ("expanded therapeutic range"), percentage of time the INR was above 5.0 or below 1.5, and number of adverse events i.e., thromboembolic and major haemorrhagic complications [18,20]. As the time interval between INR tests varies within individual patients, as well as the duration of therapy, a standard measure to indicate frequency of testing over time is number of INR tests per patient year. The number of INR tests per patient year in the PC and UC groups was reported. Guidelines suggest a minimum of four weeks between INR testing for stable patients [1], with programs often allowing a maximum time between INR testing of 6 weeks [15]. For this study, we reported the number of INR tests greater than 6 weeks apart in the PC and UC groups.

### Data analysis and sample size calculation

Based upon prior studies, it was expected that PC oral anticoagulation would result in at least a 10% absolute improvement in the percentage TTR as compared to UC [9,10]. Assuming the standard deviations of the means in the two groups are equal to 0.25 with an 80% power and a 2-tailed α of 0.05, a sample size of at least 130 patients was calculated to observe this difference in effect (65 per group). A p-value of < 0.05 was considered to be statically significant.

Statistical analysis was performed using Microsoft Excel 2003 (Microsoft Corp., Redmond, WA, USA) and SPSS statistical software for Windows (version 16.0). Patient demographics and anticoagulation control were compared using an unpaired t test, chi-squared tests, or the Fisher Exact test, as appropriate. The TTR was calculated by the ClotFree System using the method described by Rosendaal and colleagues [21].

### Ethics Approval

The Human Investigations Committee at Memorial University approved the research protocol.

## Results

The PC group included 112 patients, of which 73 had been previously managed in the UC group. The UC group included 81patients, of which eight had completed therapy with warfarin prior to the pharmacist assuming anticoagulation management. Fifty-five percent of the PC group was male with a mean age of 67 years; 51% of the UC group was male with a mean age of 71 years. The most common indications for warfarin in both groups were atrial fibrillation, mechanical heart valves and deep vein thrombosis. There was no significant difference in the demographic characteristics, indications for warfarin, or risk factors for bleeding or thromboembolic events between the PC and UC groups (Table 1).

**Table 1:** Patient Characteristics

Variable	Pharmacist Managed (PC) (n = 112)	Physician Managed (UC) (n = 81)
Age in years, mean ± SD	67 ± 18	71 ± 17
Age categories n(%):		
≤64	44 (39)	23 (28)
65 to 74	21 (19)	18 (22)
≥75	47 (42)	40 (50)

Sex, male n (%)	62(55)	41 (51)

Indication for anticoagulation, n (%)		

Atrial fibrillation	63 (56)	51 (63)

Mechanical heart valve	19(17)	17 (21)

DVT	22(20)	13(16)

PE	16(14)	8(10)

CVA	4 (3)	1 (1)

MI and/or ACS	2(2)	1 (1)

Other	1(1)	0

		

Target INR range		

2.0-3.0	93(83)	65(80)

2.5-3.5	19 (17)	16(20)

		

Risk Factors for thromboembolism or bleeding		

Hypertension	46(41)	38(47)

Previous CVA	34(30)	26(31)

Previous DVT/PE	26(23)	15 (19)

Diabetes	19(17)	9(11)

Factor II mutation	2(2)	2(3)

Factor V Leiden	7(6)	5(6)

APLS	2(2)	1(1)

ACS - acute coronary syndrome, APLS - antiphospholipid syndrome, CVA - cerebrovascular accident, DVT - deep vein thrombosis, MI - myocardial infarction, PE - pulmonary embolism

The number of INR tests per patient year in the PC and UC groups was 29 and 25, respectively. The number of INR tests per patient-year greater than 6 weeks apart in the PC and UC groups was 1.3 and 1.8, respectively (Table 2).

**Table 2:** Frequency of INR testing

Variable	Pharmacist Managed (PC) (n = 112)	Physician Managed (UC) (n = 81)
Total number of INR tests	2328	1452

Total patient years	81	59

Number of INR tests per patient year	29	25

INR tests >6weeks apart, n	103	104

Number of INR tests >6 weeks apart per patient year	1.3	1.8

The PC group spent significantly more time in both the therapeutic range (2.0-3.0 or 2.5-3.5) and the expanded therapeutic range (i.e., INR range ± 0.3) compared to the UC group. The percentage of time that the patients INR was <1.5 was less for the PC group than the UC group. The percentage of time that the patients INR was >5 was greater for the PC group than the UC group (Table 3). A subgroup analysis was conducted on the 73 patients' common to both models of care. Similar to the total sample, the PC group spent significantly more time than the UC group in the therapeutic range (71.9% vs 65.1%, p < 0.0001), as well as in the expanded therapeutic range (90.1% vs 85.0%, p < 0.0001).

**Table 3:** Anticoagulation Control

Variable	Pharmacist Managed (PC) (n = 112)	Physician Managed (UC) (n = 81)	p-value
Percentage of Time INR in Range			

Therapeutic range (TTR)*	73.4	64.8	P < 0.0001

Expanded range(Expanded TTR)†	90.8	84.8	P < 0.0001

< 1.5	0.67	1.92	P < 0.0001

>5.0	0.27	0.08	P < 0.0001

*Therapeutic range: 2.0-3.0 or 2.5-3.5; †expanded therapeutic range: therapeutic range ± 0.3

Two major bleeding events occurred in the PC group during the study period. One was an abdominal hematoma in a patient on warfarin for a mechanical heart valve. This patient had started ciprofloxacin three days prior and had an INR of 4.04 at the time of the bleed. The second event was a lower gastrointestinal (GI) bleed in a patient taking warfarin for atrial fibrillation. The GI bleed was determined to be due to diverticulosis on sigmoidoscopy. This patient had an INR of 3.16 at the time of the bleed. In both cases the bleeds were nonfatal. No major bleeding events were documented in the UC group during the study period.

One major thromboembolic event occurred in each of the PC and UC groups during the study period. The events occurred in the same patient whose warfarin care was managed by a physician during the UC timeframe and managed by the pharmacist during the PC timeframe. This patient was taking warfarin for atrial fibrillation and both events were transient ischemic attacks (TIA) requiring emergency room visits. The patient had an INR of 2.11at the time of the TIA while in the UC group and an INR of 2.44 at the time of the TIA while in the PC group.

## Discussion

The results of our study indicate that both models of care provided high quality anticoagulation management. The TTR was over 60% for both PC and UC. However, our results demonstrated that patients in the PC group spent more time in both the TTR and the expanded TTR compared to the UC group and these differences were statistically significant. The percentage of time patients' INR was < 1.5 was lower in the PC group versus the UC group and the percentage of time patients' INR was > 5 was higher in the PC group versus UC group. Low numbers of adverse clinical events were detected. Our current study's findings demonstrate that patients experience better anticoagulation control for the TTR and expanded TTR when managed by a pharmacist with expertise in anticoagulation management who applies a systematic, evidenced based approach to patient care.

Several other studies have compared pharmacist managed anticoagulation services to usual care, most often by a physician. Several of these studies support the current study findings while others indicate no difference [9-15]. The studies that support our findings include two randomized controlled trials and three observational studies [9-13]. In one Canadian study, researchers conducted a randomized controlled trial where patients were allocated to either anticoagulation clinics with a pharmacist in three tertiary hospitals (n = 112) or to their family physician practices (n = 109) [9]. Patients managed by the anticoagulation clinics were within the expanded therapeutic range more than patients managed by family physicians (82% vs 76%, p < 0.05). High risk INR values (<1.5 or >5.0) were more often observed in patients managed by family physicians (49% vs 39%, p < 0.05). In another randomized control trial conducted in Hong Kong, patients were randomized to either a pharmacist managed anticoagulation clinic (n = 68) or physician managed service (n = 69) [10]. Patients in the pharmacist managed group were within the TTR more than the physician managed group (64% vs 59%, p < 0.001). Of the three observational studies, one was conducted in Canada and two in the United States [11-13]. In one Canadian prospective cohort study, consecutive patients (n = 125) referred to the pharmacist Anticoagulation Management Service (AMS) with at least 4 months anticoagulation management prior to referral were included in a pre- and post-analysis of anticoagulation control [11]. The anticoagulation control was greater in the AMS compared with standard care in the period before referral (66.5% vs 48.8%, p < 0.0001). Both observational studies conducted in the United States found pharmacist managed anticoagulation clinics significantly improved patients anticoagulation control as measured by the TTR [12,13].

Two studies found no difference in anticoagulation control between a pharmacist managed anticoagulation clinic and usual care by a physician [14,15]. In the randomized controlled trial conducted in Quebec, patients stabilized in a pharmacist managed anticoagulation service (PMAS) in a large community hospital were subsequently randomly allocated to either continue in the PMAS (n = 128) or be transferred to their physician for follow-up care (n = 122) [14]. Researchers reported no difference in patients managed by the PMAS vs physicians in either the TTR (77.3% vs 76.7%) or expanded TTR (93.0% vs 91.6%). This finding could be explained by a potential selection bias. Patients spent an average of 11.3 weeks in the PMAS and were eligible for group allocation only if they were stable in their anticoagulation control. In addition, physicians were given the option of agreeing to participation for each patient individually or for all their patients at once. Close to 50% of the participating physicians chose patients on an individual basis to participate in the study. Consequently this study population may not have included patients with poorer anticoagulation control that would likely benefit from a pharmacist managed program. In the observational cohort study, patients in a family medicine clinic were cared for either through a pharmacist managed anticoagulation clinic or in a traditional care model by physicians [15]. Overall, the authors found no difference in anticoagulation control between the two groups. However, their method of data analysis and reporting results were not standard and make comparisons with other studies difficult.

A prospective randomized control study is generally considered the gold standard for evaluating therapeutic interventions. However it may not be the optimal method to assess this type of intervention as it is difficult to minimize observation biases. As all groups are aware of the intervention, their behaviours may be influenced accordingly in a prospective randomized trial. For example, physicians who are aware they are participating in a clinical trial may be more vigilant about monitoring anticoagulation care than in their routine practice. A well designed retrospective study may be the next best method. In our study, all eligible patients were included in the data collection, and the two groups were comparable, reducing any selection biases. This study evaluated the anticoagulation management practices in one family medicine clinic. Our patient population was similar to that in other studies evaluating anticoagulation management; consequently, our findings may be generalizable to other similar populations.

Due to the nature of the retrospective cohort study design, a number of confounders were not controlled for. The amount of time spent with patients in each model was not recorded, neither was the format or content of education provided. We did observe a subgroup of patients who crossed over from UC to PC. Although in subgroup analysis we still found a significant difference in TTR, we cannot necessarily assume this effect was due to the pharmacists' anticoagulation management. We found significant difference, but more research is needed in this area. Future research would allow for control of confounders such as time, education, protocol driven care, multivariable regression through prospective randomized controlled or prospective cohort design.

## Conclusions

The pharmacist-managed anticoagulation program within a family practice clinic compared to usual care by the physicians achieved significantly better INR control as measured by the percentage of time patients' INR values were kept in both the therapeutic and expanded range. Based on the results of this study, a collaborative family practice clinic using pharmacists and physicians may be an effective structure for an anticoagulation management service with these results verified in future prospective randomized studies.

## Competing interests

The authors declare that they have no competing interests.

## Authors' contributions

All of the authors contributed to the design of the study, the acquisition and interpretation of the data and the critical revision of the manuscript. SY contributed to the conception of the study, drafted the manuscript and is guarantor of the data. LT and SY conducted the statistical analyses of the data. All of the authors approved the final version of the manuscript submitted for publication.

## Note

Additional information about the specific protocol available from the corresponding author on request

## Pre-publication history

The pre-publication history for this paper can be accessed here:

http://www.biomedcentral.com/1471-2296/12/88/prepub

## References

[B1] Hirsh J Guyatt G Albers GW Harrington R Schünemann HJ American College of Chest Physicians Executive summary: American College of Chest Physicians Evidence- Based Clinical Practice Guidelines Chest 2008 133 8 Suppl 71 109 Erratum in: Chest 2008;134:892 10.1378/chest.08-0693 18574259

[B2] Ansell J Hirsh J Hylek E Jacobson A Crowther M Palareti G American College of Chest Physicians Pharmacology and management of the vitamin K antagonists: American College of Chest Physicians Evidence-Based Clinical Practice Guidelines (8th Edition) Chest 2008 133 Suppl 160 198 10.1378/chest.08-0670 18574265

[B3] XARELTO^®^(rivaroxaban) Product Monograph http://www.bayer.ca/files/XARELTO-PM-ENG-10SEP2008-119111.pdf?#

[B4] Pradax™ (dabigatran) Product Monograph http://www.boehringer-ingelheim.ca/content/dam/internet/opu/ca_EN/documents/humanhealth/product_monograph/Pradax-pm.pdf

[B5] Patel MR Mahaffey KW Garg J Pan G Singer DE Hacke W Breithardt G Halperin JL Hankey GJ Piccini JP Becker RC Nessel CC Paolini JF Berkowitz SD Fox KA Califf RM the ROCKET AF Steering Committee for the ROCKET AF Investigators Rivaroxaban versus Warfarin in Nonvalvular Atrial Fibrillation N Engl J Med 2011 http://www.nejm.org/doi/full/10.1056/NEJMoa1009638

[B6] Connolly SJ Ezekowitz MD Yusuf S Eikelboom J Oldgren J Parekh A Pogue J Reilly PA Themeles E Varrone J Wang S Alings M Xavier D Zhu J Diaz R Lewis BS Darius H Diener HC Joyner CD Wallentin L RE-LY Steering Committee and Investigators Dabigatran versus warfarin in patients with atrial fibrillation N Engl J Med 2009 361 1139 1151 10.1056/NEJMoa0905561 19717844

[B7] Buller HR Lensing AW Prins MH Agnelli G Cohen A Gallus AS Misselwitz F Raskob G Schellong S Segers A Einstein-DVT Dose-Ranging Study investigators A dose-ranging study evaluating once-daily oral administration of the factor Xa inhibitor rivaroxaban in the treatment of patients with acute symptomatic deep vein thrombosis: the Einstein- DVT Dose-Ranging Study Blood 2008 112 2242 2247 10.1182/blood-2008-05-160143 18621928

[B8] Schulman S Kearon C Kakkar AK Mismetti P Schellong S Eriksson H Baanstra D Schnee J Goldhaber SZ RE-COVER Study Group Dabigatran versus warfarin in the treatment of acute venous thromboembolism N Engl J Med 2009 361 2342 2352 10.1056/NEJMoa0906598 19966341

[B9] Wilson SJ Wells PS Kovacs MJ Lewis GM Martin J Burton E Anderson DR Comparing the quality of oral anticoagulant management by anticoagulation clinics and by family physicians: a randomized controlled trial CMAJ 2003 169 293 298 Erratum in: CMAJ 2004,170:451 12925422 PMC180652

[B10] Chan FW Wong RS Lau WH Chan TY Cheng G You JH Management of Chinese patients on warfarin therapy in two models of anticoagulation service - a prospective randomized trial Br J Clin Pharmacol 2006 62 601 609 10.1111/j.1365-2125.2006.02693.x 17061966 PMC1885165

[B11] Bungard TJ Gardner L Archer SL Hamilton P Ritchie B Tymchak W Tsuyuki RT Evaluation of a pharmacist-managed anticoagulation clinic: Improving patient care Open Med 2009 3 e16 21 19946388 PMC2765765

[B12] Rudd KM Dier JG Comparison of two different models of anticoagulation management services with usual medical care Pharmacotherapy 2010 30 330 338 10.1592/phco.30.4.330 20334453

[B13] Chiquette E Amato MG Bussey HI Comparison of an anticoagulation clinic with usual medical care: anticoagulation control, patient outcomes, and health care costs Arch Intern Med 1998 158 1641 1647 10.1001/archinte.158.15.1641 9701098

[B14] Lalonde L Martineau J Blais N Montigny M Ginsberg J Fournier M Berbiche D Vanier MC Blais L Perreault S Rodrigues I Is long-term pharmacist-managed anticoagulation service efficient? A pragmatic randomized controlled trial Am Heart J 2008 156 148 154 10.1016/j.ahj.2008.02.009 18585510

[B15] Chamberlain MA Sageser NA Ruiz D Comparison of anticoagulation clinic patient outcomes with outcomes from traditional care in a family medicine clinic J Am Board Fam Pract 2001 14 16 21 11206689

[B16] Farrell B Pottie K Woodend K Yao V Dolovich L Kennie N Sellors C Shifts in expectations: evaluating physicians' perceptions as pharmacists become integrated into family practice J Interprof Care 2010 24 80 9 10.3109/13561820903011968 19705320

[B17] Ansell JE Buttaro ML Thomas OV Knowlton CH Consensus guidelines for coordinated outpatient oral anticoagulation therapy management. Anticoagulation Guidelines Task Force Ann Pharmacother 1997 31 604 615 9161658 10.1177/106002809703100516

[B18] Phillips KW Ansell J Outpatient management of oral vitamin K antagonist therapy: defining and measuring high-quality management Expert Rev Cardiovasc Ther 2008 6 57 70 10.1586/14779072.6.1.57 18095907

[B19] Statistics Canada Population of census metropolitan areas (2006 Census boundaries) http://www40.statcan.gc.ca/l01/cst01/demo05a-eng.htm

[B20] Schulman S Kearon C Subcommittee on Control of Anticoagulation of the Scientific and Standardization Committee of the International Society on Thrombosis and Haemostasis Definition of major bleeding in clinical investigations of antihemostatic medicinal products in non-surgical patients J Thromb Haemost 2005 3 692 694 10.1111/j.1538-7836.2005.01204.x 15842354

[B21] Rosendaal FR Cannegieter SC van der Meer FJ Briët E A method to determine the optimal intensity of oral anticoagulant therapy Thromb Haemost 1993 69 236 239 8470047

